# A New View of Alcohol Metabolism and Alcoholism—Role of the High-*K_m_* Class III Alcohol Dehydrogenase (ADH3)

**DOI:** 10.3390/ijerph7031076

**Published:** 2010-03-15

**Authors:** Takeshi Haseba, Youkichi Ohno

**Affiliations:** Department of Legal Medicine, Nippon Medical School, 1-1-5 Sendagi, Bunkyo-ku, Tokyo 113-8602, Japan; E-Mail: ohno@nms.ac.jp

**Keywords:** ADH1, ADH3, contribution to alcohol metabolism, enzyme regulation, role shift, alcoholic liver disease

## Abstract

The conventional view is that alcohol metabolism is carried out by ADH1 (Class I) in the liver. However, it has been suggested that another pathway plays an important role in alcohol metabolism, especially when the level of blood ethanol is high or when drinking is chronic. Over the past three decades, vigorous attempts to identify the enzyme responsible for the non-ADH1 pathway have focused on the microsomal ethanol oxidizing system (MEOS) and catalase, but have failed to clarify their roles in systemic alcohol metabolism. Recently, using ADH3-null mutant mice, we demonstrated that ADH3 (Class III), which has a high *K_m_* and is a ubiquitous enzyme of ancient origin, contributes to systemic alcohol metabolism in a dose-dependent manner, thereby diminishing acute alcohol intoxication. Although the activity of ADH3 toward ethanol is usually low *in vitro* due to its very high *K_m_*, the catalytic efficiency (*k_cat_*/*K_m_*) is markedly enhanced when the solution hydrophobicity of the reaction medium increases. Activation of ADH3 by increasing hydrophobicity should also occur in liver cells; a cytoplasmic solution of mouse liver cells was shown to be much more hydrophobic than a buffer solution when using Nile red as a hydrophobicity probe. When various doses of ethanol are administered to mice, liver ADH3 activity is dynamically regulated through induction or kinetic activation, while ADH1 activity is markedly lower at high doses (3–5 g/kg). These data suggest that ADH3 plays a dynamic role in alcohol metabolism, either collaborating with ADH1 or compensating for the reduced role of ADH1. A complex two-ADH model that ascribes total liver ADH activity to both ADH1 and ADH3 explains the dose-dependent changes in the pharmacokinetic parameters (β, CL_T_, AUC) of blood ethanol very well, suggesting that alcohol metabolism in mice is primarily governed by these two ADHs. In patients with alcoholic liver disease, liver ADH3 activity increases, while ADH1 activity decreases, as alcohol intake increases. Furthermore, ADH3 is induced in damaged cells that have greater hydrophobicity, whereas ADH1 activity is lower when there is severe liver disease. These data suggest that chronic binge drinking and the resulting liver disease shifts the key enzyme in alcohol metabolism from low-*K_m_* ADH1 to high-*K_m_* ADH3, thereby reducing the rate of alcohol metabolism. The interdependent increase in the ADH3/ADH1 activity ratio and AUC may be a factor in the development of alcoholic liver disease. However, the adaptive increase in ADH3 sustains alcohol metabolism, even in patients with alcoholic liver cirrhosis, which makes it possible for them to drink themselves to death. Thus, the regulation of ADH3 activity may be important in preventing alcoholism development.

## 1. Introduction

The pharmacologic and potentially pathologic effects of alcohol depend on the concentrations of ethanol and its metabolites in the body, and on the duration of exposure to these substances. When the body ingests ethanol, there is an initial equilibration phase, after which both the concentrations and duration of these substances in the body are primarily governed by the rate of ethanol metabolism. So, the elucidation of the mechanism of alcohol metabolism is essential to the understanding and control of the action of alcohol in the body.

Alcohol dehydrogenase (ADH) 1 (Class I ADH) is the key enzyme in alcohol metabolism *in vivo* [1]. However, it has been demonstrated that systemic alcohol metabolism involves another pathway independent of ADH1 [2,3]. This was originally called the non-ADH1 pathway, and is thought to play a major role in alcohol metabolism for acute intoxication and for chronic drinkers [2–7]. Thus, the identification of the enzyme in this pathway has long been the subject of heated scientific debate, with the main candidates being the microsomal ethanol oxidizing system (MEOS) and catalase [2–12]. However, the contributions of these two enzymes to systemic alcohol metabolism have still not been clarified.

In 1978, we found a new type of ADH in mouse liver [13,14], which was later classified as Class III ADH (ADH3) [14,15], and have continued to investigate its enzymatic characteristics and its role in alcohol metabolism [16–24]. Recently, we used ADH3-null mutant mice to demonstrate that the contribution of ADH3 to alcohol metabolism *in vivo* increases dose-dependently [25].

In this review, we argue for a new view of ADH3 in alcohol metabolism and in the development of alcoholism, and compare it with ADH1, which has the starring role in the field of alcohol medicine.

## 2. Historical Debate on the Identification of Enzyme(s) in the So-Called ‘Non-ADH’ Pathway* of Alcohol Metabolism

Animal experiments using pyrazoles, which are specific inhibitors of ADH, have left no doubt that ADH1 is the main enzyme responsible for alcohol metabolism in the body [1]. However, alcohol continues to be metabolized to some extent even after the administration of pyrazoles; this is called the non-ADH pathway [2,3]. This pyrazole-insensitive pathway has a greater metabolic role when the level of blood alcohol is high or when the intake of alcohol is chronic [2–7]. Recently, we used ADH1-null mice to show that ADH1 accounts for about 70% of systemic alcohol metabolism [25,26], which means that the non-ADH1 pathway accounts for the remaining 30%. Regarding the identity of the main enzyme responsible for this pathway, a heated scientific debate has continued for three decades over whether it is catalase [10–12] or MEOS, which is mainly composed of CYP2E1 [2–9]. Indeed, both enzymes seem to be responsible for the non-ADH1 pathway, because both exhibit ethanol-oxidizing activity with a *K_m_* for ethanol (around 10 mM [7,10]) that is higher than that for ADH1, and both are insensitive to pyrazoles *in vitro* [2–6]. Furthermore, the induction of MEOS activity due to chronic alcohol consumption seems to explain the accelerated rate of alcohol metabolism observed in chronic drinkers [3,5–7]. However, it has recently been demonstrated that there is no difference in the elimination rate for blood ethanol between CYP2E1-null mice and wild-type mice, even after the chronic administration of ethanol [27]. The role of catalase in systemic alcohol metabolism has not been clarified *in vivo* either, even though mice genetically lack this enzyme [28]. Since there is no *in-vivo* evidence for either MEOS or catalase, the debate still has not reached a conclusion. Furthermore, the first-order kinetics of the elimination of blood alcohol has been observed at very high concentrations [29–31], which cannot be explained by the *K_m_* of either MEOS [7] or catalase [10]. These data suggest the involvement of a very high-*K_m_* enzyme in the non-ADH pathway.

Many investigators have reported that the total ADH activity of liver correlates with the rate of systemic alcohol metabolism [1]. Mammalian livers are known to contain two ADH isozymes other than ADH1, namely, ADH2 (Class II) and ADH3 (Class III), both of which have a higher *K_m_* for ethanol than ADH1 [32]. These data suggest a possible role for these high-*K_m_* ADHs in the non-ADH1 pathway of alcohol metabolism.

## 3. Discovery of ADH3 (Class III ADH)

In 1981, X. Pares and B. L. Vallee reported a new form of human liver ADH with unique kinetic characteristics [33] and classified it as Class III ADH (ADH3) because it was the third isozyme of mammalian ADH to be discovered [15]. However, two years earlier in 1979, at a meeting on alcohol medicine in Japan [13], we reported that we had found a new type of ADH with an acidic isoelectric point in mouse liver that had a very high *K_m_* for ethanol and was insensitive to pyrozoles. The new mouse ADH corresponded to an electrophoretic ADH band previously designated as ADHB_2_ on a gel [14,34] and was later also classified as Class III ADH (ADH3) [14,33]. Around that time, many investigators considered ADH3 to have a negligible role in alcohol metabolism because it exhibits only very low activity towards ethanol due to its very high *K_m_* when measured *in vitro* by the standard ADH assay using 15 mM ethanol as a substrate [33]. However, when the ADH3 fraction was separated from other ADH isozymes of mouse liver by CM-chromatography, it was found to account for about 13% of total liver ADH activity; ADH1 accounts for more than 62%, and ADH2 accounts for only 2%. Moreover, as the concentration of substrate ethanol increased, the percentage of the ADH3 fraction increased while that of the ADH1 fraction decreased. So, we speculated that ADH3 plays a significant role in alcohol metabolism [24].

## 4. Activation of ADH3 by Solution Hydrophobicity and Dynamic Metabolism of Alcohol

Mouse ADH1 shows the largest activity towards ethanol around 10 mM (*K_m_* ≒ 1 mM), and the activity decreases in a dose-dependent manner as the concentration becomes higher. In contrast, ADH3 shows a low activity towards ethanol at several tens of millimoles, but the activity increases linearly up to the molar level (*K_m_* < 1 M) [15,16]. However, we have sometimes observed that ADH3 is activated during the purification process when measured by the standard ADH assay. By investigating the regulation of its activity under various conditions, we found that solution hydrophobicity markedly activated ADH3 and increased its catalytic efficiency (*k_cat_*/*K_m_*) for ethanol [20,25]. Tert-butanol (C4), which strongly induces solution hydrophobicity in a medium due to its strong hydrophobic hydration and its high miscibility, increases the *k_cat_*/*K_m_* of ADH3 almost ten-fold, lowering its *K_m_* for ethanol. The *K_m_* decreases below 100 mM by solution hydrophobicity induced with tert-butanol above 1.8 M (unpublished data). Since we observed no significant changes in the secondary, tertiary, or quaternary structures of mouse ADH3 in hydrophobic solution, we concluded that solution hydrophobicity induces a slight structural change in the substrate-binding pocket of ADH3 [25]. Indeed, the reason why ADH3 has a low activity for ethanol is that the volume of its substrate-binding pocket is larger than that of ADH1 due to the substitution of smaller, more hydrophilic amino acid residues [35]. The hydrophilic amino acids constituting the pocket of ADH3 collapse in a hydrophobic medium, thus, reducing the size of the pocket. This structural change may raise its affinity for a small molecule like ethanol, thereby increasing its ability to oxidize ethanol. Thus, solution hydrophobicity lowers the *K_m_* of ADH3 and increases it activity with respect to ethanol. The increase in activity in a hydrophobic solution is specific to ADH3; all other ADH isozymes show a decrease in activity in this type of medium [20].

In contrast to water molecules *in vitro*, intracellular ones are dynamically restrained by their hydrophobic interaction with various macromolecules and intracellular membranes with an extensive reticulum [36]. When Nile red, a fluorescent probe for hydrophobicity, is added to the cytoplasm of mouse liver, its spectrum is red-shifted from the spectrum obtained when it is added to a lipid droplet or a membrane of the liver, reflecting the hydrophobicity of a cytoplasmic solution [25]. Thus, also *in vivo*, ADH3 should be activated and ADH1 activity should be depressed by solution hydrophobicity in the liver cells. It is reasonable to conclude that both ADHs have dynamic roles in alcohol metabolism, with their activities being inversely regulated by changes in the solution hydrophobicity of liver cells.

## 5. Role of ADH3 in Systemic Alcohol Metabolism and in the Action of Alcohol in the Body—*in vivo* Evidence from ADH-knockout Mice

In order to clarify the roles of ADH1 and ADH3 in systemic alcohol metabolism and in the action of alcohol in the body at various ethanol doses, we investigated the rate of alcohol metabolism (EDR: mg/kg/h) in wild-type mice (*Wild*), ADH1-null mutant mice (*Adh1-/-*) and ADH3-null mutant mice (*Adh3-/-)* by the administration of ethanol at doses ranging from 1.0 to 4.5 g/kg [25]. Compared to the EDR of *Wild*, that of *Adh3-/-* decreased markedly as ethanol dose increased and was about 66% of that of *Wild* at a dose of 4.5 g/kg. These data demonstrate that ADH3 contributes to systemic alcohol metabolism in a dose-dependent manner *in vivo*, with the contribution being over 30% at large doses. These data also indicate that the contribution of ADH1 to alcohol metabolism decreases dose-dependently because ADH1 is the only major alcohol-metabolizing enzyme in *Adh3-/-*. In addition, the EDR of *Adh1-/-* was 21–33% that of *Wild*, which indicates that the contribution of ADH1 to systemic alcohol metabolism is more than 60% but less than 80%, and decreases dose-dependently. Thus, we conclude that the contribution of ADH3 to alcohol metabolism increases as the dose increases, thereby compensating for the reduced role of ADH1. Moreover, when the ethanol dose is large, ADH1 and ADH3 together account for almost all the alcohol metabolism of *Wild*.

The duration of the loss of the righting reflex (LORR) in mice, which is the length of time that a mouse sleeps after the administration of enough ethanol to produce acute intoxication, was longer in the order *Adh1-/-*, *Adh3-/-*, and *Wild*. The frequency of mortality at large doses also increased in the order *Adh1-/-*, *Adh3-/-*, and *Wild*. These data demonstrate that, *in vivo,* ADH3 diminishes acute alcohol intoxication together with ADH1, by participating in alcohol metabolism in a dose-dependent manner.

## 6. The Role of ADH3 in First-Pass Metabolism

After drinking alcohol, it is partially metabolized in the digestive organs including the liver, before entering the systemic circulation of blood. The concentration of alcohol reaches a molar level in these organs. It is possible for ADH3 to play an important role in this first-pass metabolism (FPM) because it has a very high *K_m_* for ethanol and is present not only in the liver but also in the stomach and small intestine. Indeed, the ADH-3 activity in the stomach and the FPM are smaller for women than for men [37]. Furthermore, we found that, in mouse liver, ADH3 highly distributes in sinusoidal endothelial cells, in contrast to ADH1, which is present only in parenchymal cells [19]. So, it seems that, when a high concentration of alcohol is carried from a portal vein to the liver, it is first metabolized by high-*K_m_* ADH3 in sinusoidal endothelial cells; and then, after ADH3 has lowered the concentration, the remained ethanol is metabolized by low-*K_m_* ADH1 in parenchymal cells. Thus, the FPM of alcohol in the liver may involve a two-step process employing both ADHs, which have different *K_m_*s for ethanol and different tissue distributions. ADH3 may play the role of a gatekeeper that protects the parenchymal cells from the high toxicity of high concentrations of alcohol.

## 7. Dynamic Contribution of ADH3 to Alcohol Metabolism through Regulation of Activity by Ethanol Administration

When a small dose (1 g/kg) of ethanol is administered to mice, the blood alcohol concentration (BAC) reaches around 20 mM, at which ADH1 shows the maximum activity. This level of BAC is also observed in people who become slightly drunk after imbibing about two 633-mL bottles of beer. A medium dose (3 g/kg) causes mice to lose the righting reflex for several minutes just after administration, and the BAC is around 60 mM. A large dose (5 g/kg) produces acute alcohol intoxication with a BAC of over 100 mM, and the righting reflex is lost for several hours [24,25].

To investigate how the activities of ADH1 and 3 change during alcohol metabolism and what their relative contributions are, we periodically measured total liver ADH activity and the content of the two ADHs after various doses of ethanol were administered to mice [24]. When measured by the standard ADH assay using 15 mM ethanol as a substrate, the total liver ADH activity involved not only ADH1 but also ADH3, from the data that the total activity correlates more strongly with the sum of two ADH contents than with just the ADH1 content. When the ethanol dose was small, the contents of both ADHs in the liver increased, resulting in about a 41% increase in total liver ADH. Thus, both ADHs are acutely induced in the liver and actively participate in alcohol metabolism at moderate drinking. The acute induction of ADH isozymes, which are due to increases in mRNA [38], are known to occur in yeast [39] and plant [40] cells in hypoxic conditions. These data suggest that the acute induction of liver ADHs observed in mouse by a small dose of ethanol is also due to hypoxic conditions of the liver cell, where NADH/NAD ratio is increased by ethanol metabolism [41]. ADH3 may be related to brain disorders due to hypoxia, because it distributes in the brain in areas that are vulnerable to ischemia or hypoxic cerebropathy [23] as the sole ADH isozyme in the brain [42]. When the ethanol dose was medium [24], the liver ADH1 content decreased from the early stages to the end of alcohol metabolism. On the other hand, the liver ADH3 content increased in the early stages, but decreased in later stages, which is similar to the time-wise change found in the total ADH activity of the liver. For a large dose [24], the content of both ADHs in the liver markedly decreased, starting just after ethanol administration, which resulted in a marked decrease in total ADH activity. However, the ADH3 content recovered and increased in the later stages of alcohol metabolism, although that of ADH 1 kept decreasing to the end. Thus, at intoxicating doses of ethanol, ADH3 makes a positive contribution to alcohol metabolism, as shown by the time-wise change in its content in the liver during alcohol metabolism, whereas the contribution of ADH 1 decreases because its content in the liver decreases.

Another point is that the ADH3 activity of liver extract can be measured by using hexenol as a substrate and 4-methylpyrazole to inhibit ADH1 [43]. Even though the ADH3 content exhibited a time-dependent change at intoxicating doses of ethanol (3, 5 g/kg), the ADH3 activity assayed by this method remained high during alcohol metabolism, as observed for the small dose [44]. Furthermore, the catalytic efficiency (*V_max_*/*K_m_*) of the ADH activity of liver extract for ethanol increased dose-dependently [45]. These data suggest that at intoxicating doses of ethanol, ADH3 activity remains high by regulation of both enzyme composition and enzyme kinetics to compensate for the reduced role of ADH1 in alcohol metabolism. Thus, both ADH1 and ADH3 contribute to alcohol metabolism, dynamically changing the enzyme composition and kinetics.

## 8. A Complex Two-ADH Model to Explain Pharmacokinetics of Blood Alcohol

In mice [24,45], the alcohol clearance (CL_T_: dose/AUC) decreased in a dose-dependent manner up to a dose of 5 g/kg, and exhibited a significant correlation with average total liver ADH activity during alcohol metabolism. The elimination rate for blood alcohol (β) reached a maximum at an ethanol dose of 2 g/kg, which corresponds to the *V_max_* of Michaelis-Menten (M-M) elimination kinetics. However, above a dose of 2 g/kg, β decreased as the dose increased, as CL_T_ decreased. The decrease in β above a medium dose has also been observed in rats [46,47]. On the other hand, the normalized AUC (AUC/dose), which exhibited a nonlinear, dose-dependent increase, had an inverse correlation with β.

To explain how alcohol-metabolizing enzymes regulate the pharmacokinetics of blood alcohol in mice, we have devised a complex two-ADH model in which the total liver ADH activity involves not only ADH1 but also ADH3 [24,45]. According to the model, liver ADH activity is a function of four variables: ADH1 activity, ADH1 content, ADH3 activity, and ADH-3 content. Since liver ADH3 activity is determined not only by the change in content but also by the change in kinetics, as mentioned in the previous section, we make the following definitions:

liver ADH3 activity = total liver ADH activity – liver ADH1 activity, and
liver ADH1 activity = liver ADH1 content × *V_max_*/mg for purified ADH1.

When the activities of both ADHs are normalized by the ratio of the liver ADH activity of mice to which ethanol was administered mice to that of control mice, the ADH3 activity increased dose-dependently, whereas that of ADH1 decreased. The ADH3/ADH1 activity ratio correlates directly with AUC/dose, and inversely with CL_T_ and β. So, the dose-dependent increase in AUC is explained by the dose-dependent increase in the ADH3/ADH1 activity ratio, which causes the body clearance (CL_T_) and elimination rate (β) of alcohol metabolism to decrease.

Thus, the complex two-ADH model explains the changes in various pharmacokinetic parameters of blood alcohol in mice, suggesting that, as the ethanol dose increases, the elimination kinetics of blood alcohol shift from M-M kinetics dominated by ADH1 to first-order kinetics dominated by ADH3 [45]. The first-order elimination kinetics of blood ethanol has been observed in humans at blood ethanol levels above 100 mM [29,30]. As the ethanol dose increases, the shift in enzyme from a low-*K_m_* ADH to a high-*K_m_* ADH causes a nonlinear increase in AUC due to the lowered metabolic rate while the ability of alcohol to be metabolized is maintained. These metabolic changes may qualitatively enhance the toxicity of alcohol.

## 9. Regulation of ADH3 Activity in Chronic Ethanol Feeding and its Role in Alcohol Metabolism

Animal experiments on chronic ethanol feeding have usually been performed on rats using a liquid diet (Lieber diet) containing 5% ethanol, which corresponds to 36% of the total caloric intake. In these experiments, liver MEOS shows increased activity, whereas liver ADH shows reduced or unchanged activity after four weeks of feeding, which accelerates alcohol metabolism and causes a severe case of fatty liver [7]. Based on these data, it has been thought that the acceleration of alcohol metabolism in chronic drinking is due to the induction of MEOS. However, it has recently been shown that there is no difference in the elimination rate for blood ethanol between CYP2E1 (a major component of MEOS)-null mice and wild-type mice, even after chronic ethanol feeding [27]. These data show that the acceleration of alcohol metabolism caused by chronic ethanol feeing is not related to MEOS.

We investigated the effects of chronic ethanol feeding on liver ADH isozymes under milder conditions than usual, using mice with about twice the rate of alcohol metabolism as that of rats and a liquid diet containing 4% ethanol, which corresponds to 28% of the total caloric intake [48]. After one week of feeding, the rate of alcohol metabolism increased as the total liver ADH activity increased, which was due to an increase in the liver ADH1 content. This situation continued until the 24th week of feeding, when a moderate case of fatty liver was observed. So, it seems that ADH1 adaptively contributes to the acceleration of alcohol metabolism induced by chronic ethanol feeing until the liver becomes moderately fatty, but that the contribution decreases as liver disease progresses, as observed in rats with a severe case of fatty liver.

On the other hand, the liver ADH3 content markedly increased with the acute administration of a large dose (4.5 g/kg) of ethanol to mice during chronic ethanol feeding, although the liver ADH3 content did not significantly change when chronic ethanol feeding was mild [48]. This phenomenon may be related to acute alcoholic hepatitis or pancreatitis. It has been suggested that only ADH3 metabolizes ethanol in pancreatic acinar cells, which have almost the same ability to metabolize high concentrations of alcohol (50 mM) as hepatocytes do [49]. This also suggests that ADH3 is related to alcoholic pancreatic disease.

## 10. Induction of ADH3 Due to Cell Damage Accompanied by Increased Hydrophobicity

ADH3 is strongly expressed in human atherosclerotic lesions of the coronary artery and in cultured rat smooth-muscle cells after lipofection, in both of which membrane damage and a hydrophobic state were observed [22]. These data suggest that ADH3 is induced by cell damage accompanied by increased hydrophobicity. So, the increase in liver ADH3 content observed in mice in the late stages of alcohol metabolism after the administration of a large dose of ethanol (Section 6) may be due to the cell damage and fatty change, which are observed in mouse liver late after a large dose of ethanol [50]. The marked increase in liver ADH3 content observed after the acute administration of a large dose of ethanol to chronic-ethanol-fed mice (Section 8) might have the same cause. Thus, the activity of ADH3 in the cells seems to be dynamically regulated by various intracellular conditions, for example, at the kinetic level, by solution hydrophobicity (Section 5), and at the enzyme content level, by hypoxia (Section 6) or by cell damage accompanied by increased hydrophobicity. ADH3 tends to be expressed especially strongly in cells in a critical condition because it plays a fundamental role in cell functions as the evolutionary ancestor of ADH, in contrast to ADH1, which differentiated from ADH3. The chronic intake of a large amount of alcohol forces to enhance ADH3 activity, but suppress ADH1 activity, through increased solution hydrophobicity and cell damage. Furthermore, ADH3 activity may be elevated throughout the body of alcoholics because it is ubiquitous. Thus, ADH 3 may play a greater role in the alcohol metabolism of alcoholics.

## 11. Role of ADH3 in Liver Disease and the Alcohol Metabolism of Alcoholics

When hexenol is used as a substrate in an ADH assay, 4-methlypyrazole has no effect on ADH3 activity, but it completely inhibits ADH1 activity [43]. So, we used hexenol and 4-methlypyrazole to measure the liver ADH1 and ADH3 activities of alcoholics with liver disease [18]. The total ADH and ADH1 activities of biopsied livers correlate directly with each other and inversely with the amount of alcohol intake per day. These activities are relatively high in normal livers and in livers with mild fibrosis, but are low in fatty livers and very low in livers with severe fibrosis and cirrhosis. Taking these results together with those for chronically-ethanol-fed mice (Section 8), we conclude that chronic drinking adaptively increases ADH1 activity to accelerate alcohol metabolism until the onset of mild liver disease. However, a further increase in alcohol intake reduces ADH1 activity and its role in alcohol metabolism due to the development of liver disease. On the other hand, ADH3 activity significantly correlates with a person’s total alcohol intake, which strongly correlates with the development of liver disease. Consequently, ADH3 activity inversely correlates with ADH1 activity in alcoholic patients. This relationship can be also explained by the idea that ADH 3 is induced by cell damage accompanied by increased hydrophobicity. In addition, capillarization of the liver due to liver cirrhosis may also increase the amount of ADH3, because it is mainly found in the sinusoidal endothelial cells of the liver [19]. The highest incidence of liver cirrhosis is observed in patients with a high ADH3/ADH1 activity ratio plus low total liver ADH activity. As discussed in the complex two-ADH model for mice, the key enzyme in alcohol metabolism may also shift from ADH1 to ADH3 in alcoholic patients with severe liver disease. For these patients, liver disease may have developed through a nonlinear increase in AUC due to an increase in the ADH3/ADH1 activity ratio that accompanies the lower rate of alcohol metabolism.

Cases are not rare in which alcoholics with liver cirrhosis visit a hospital after drinking a great deal of alcohol the day before. How can they drink so much even though ADH1, MEOS [51], and catalase [52], which are well-known alcohol-oxidizing enzymes, all have a low activity in their livers? It is difficult to explain the alcohol metabolism of such patients solely in terms of these enzymes. On the other hand, ADH3 is induced by cell damage that produces hydrophobicity [22] and positively correlates with lifetime total alcohol intake [18], which is strongly related to the development of liver disease. So, this ADH may maintain the ability of alcoholic patients to metabolize alcohol in spite of severe liver disease, thereby making it possible for them to drink a great deal.

In summary, the development of alcoholism is adequately described by a complex two-ADH model. Chronic drinking accelerates alcohol metabolism by means of an adaptive increase in ADH1 activity (and also ADH3 activity under some conditions) during the non-pathological stage. Induced alcohol resistance increases the amount of alcohol that can be imbibed, and leads to heavy drinking. The increased AUC from heavy drinking elevates the cellular toxicity and central-nervous-system toxicity of alcohol. Cell damage accompanied by hydrophobicity reduces the rate of alcohol metabolism through an increase in the ADH3/ADH1 activity ratio, which in turn produces a further nonlinear increase in AUC. The vicious cycle of an interdependent increase in the ADH3/ADH1 activity ratio and AUC in alcohol dependence may further develop liver disease and induce cirrhosis of the liver. However, this adaptation of ADH3 to alcohol metabolism may mean suicide to alcoholics, because it makes it possible for them to keep on drinking by maintaining the ability to metabolize alcohol even when there is severe liver disease, which could be fatal. This seems to be an adaptive discrepancy in human physiology. From another standpoint, however, the regulation of ADH3 activity seems to be a key to resolving the problem of alcoholism.

## 12. Other ADH Isozymes in Alcohol Metabolism

ADH2 (Class II), which shows limited distribution to the liver [53], seems to be insignificant in alcohol metabolism of rodents, because mouse ADH2 is responsible for only a small part of the total ADH activity in the liver (Section 3). On the other hand, human ADH2 is known to be highly active towards ethanol [54], and is suggested to contribute to alcohol metabolism [55,56]. However, its contribution possibility to systemic alcohol metabolism may become lower in habitual drinking, because ADH2 activity has been demonstrated to be decreased in baboon liver by moderate drinking and to be almost abolished by heavy drinking [57].

ADH4 (Class IV) also shows restricted distribution to gastric mucosa, exhibiting different kinetics for ethanol between human (K_m_ = ~37 mM) and rodents (K_m_ = ~2.4 M) [58]. The catalytic efficiency is about 40-times higher in human than in rodent. However, the tissue content of human ADH4 in gastric mucosa is approximately 10 μg/g tissue [58], which is less than 1/40 of the contents of ADH1 or 3 in the liver [20,59]. Moreover, the contribution of the stomach to first pass metabolism (FPM) is known to be much smaller than the liver [55]. Therefore, the involvement of ADH4 in alcohol metabolism may be small even in FPM.

## 13. Physiological Roles of ADH3 and Alcohol-Related Diseases

It goes without saying that ADHs essentially exist in human body to carry out physiological metabolisms, aside from their ability to metabolize the alcohol that a person imbibes. In fact, they play important roles in the metabolism of physiological alcohol and aldehyde derivatives, such as neurotransmitters with active amines (dopamine [60], norepinephrine [61], serotonin [62] *etc.*), cholesterols [63], steroids [64,65], bile acids [66], and Vitamin A [67]. ADH 3, in particular, is found in all living organisms as the ancestor of other forms of ADH, and is considered to play an important role in basic cell metabolism [68]. For example, ADH3 is identical to glutathione-dependent formaldehyde dehydrogenase (GSH/FADH) [69], which is the enzyme responsible for detoxifying formaldehyde in the body [26]. In addition, the s-nitrosoglutathion (GSNO) reductase activity of ADH3 plays a key role in NO metabolism [70]. ADH3 may also be involved in anti-inflammatory, anti-allergic reactions and blood pressure by metabolizing 20-OH leukotriene B4 [71] and other ω-OH fatty acids [72]. Moreover, all ADH isozymes oxidize retinol (Vitamin A), and ethanol strongly inhibits this activity [73]. Deltour *et al*. considered the inhibition of retinoic acid synthesis by ethanol to be a potential mechanism for fetal alcohol syndrome (FAS) [74]. Retinoic acid, the metabolite of Vitamin A, plays an important role in the neural development of vertebrates [75]. Molotkov *et al.* have demonstrated that ADH3 is a key enzyme in the systemic metabolism of Vitamin A, although it shows little activity for Vitamin A *in vitro* [67]. It also regulates the content of retinoic acid in the brain, because it is the sole ADH in the brain [23,42]. So, it seems that FAS, which is accompanied by various neuronal abnormalities, arises because ethanol inhibits retinol metabolism through ADH3 inhibition in an embryo or a fetus brain. Thus, ADH3 is a multifunctional house-keeping enzyme with a wide specificity for substrates and is responsible for various metabolic functions essential for life, including the detoxification of cytotoxins. So, it is easy to imagine that, in heavy drinkers, the continuous drinking of alcohol disturbs the basic metabolism and causes the collapse of homeostasis by inhibiting the many functions of ADH3. The idea that alcohol-related disorders are induced when ethanol inhibits physiological metabolism through the mechanism of ADHs is as important as ideas on the toxicity of ethanol and its metabolites themselves.

## 14. Conclusion

This review described a new view of ADH 3 in the fields of alcohol metabolism and the bioaction of ethanol, and compared the new face of ADH3 to the well-known ADH1. ADH 3 contributes to alcohol metabolism during acute alcohol intoxication and diminishes the symptoms, thereby supporting ADH1. Solution hydrophobicity increases ADH3 activity at the kinetic level, and cell damage accompanied by hydrophobicity does so at the enzyme content level. A dose of alcohol large enough to damage the liver increases the ADH3/ADH1 activity ratio and lowers the rate of alcohol metabolism, which in turn nonlinearly increases the AUC. The interdependent increase in AUC and the ADH3/ADH1 activity ratio may be a factor in the development of liver disease. In alcoholics with liver disease, ADH3 activity increases with total alcohol intake, while ADH1 activity decreases. This suggests that ADH3 plays an important role in the alcohol metabolism of alcoholics with severe liver disease, compensating for the reduced role of ADH1. This adaptive role of ADH3 after chronic binge drinking makes it possible for alcoholics to drink themselves to death in spite that the rate of alcohol metabolism is lowered. Thus, it may be important to regulate ADH3 activity to prevent the development of alcoholism.

Our theory about alcohol metabolism and alcoholism is still at the research level. We welcome a thorough examination of it through experiments and discussions in various fields.

## Footnotes

* The term “non-ADH pathway” was invented when only one mammalian ADH isozyme was known to exist, namely, Class I ADH (ADH1). Today, we know of six mammalian ADH isozymes; classified as Class I to VI in order of discovery [76]. Class I, II, III, and IV ADHs are present in every mammal and are called ADH 1, 2, 3 and 4, respectively. Accordingly, the term “non-ADH pathway” actually means the non-ADH1 pathway. In humans, ADH1 is further classified into three subcategories: ADH1A (formerly ADH1), 1B (formerly ADH2) and 1C (formerly ADH3), which contain a homodimer of subunit α, β, and γ, respectively. The amino acid sequences for the three ADH1 isozymes are about 91% homologous [77]. On the other hand, ADH2 (formerly ADH4), 3 (formerly ADH5), and 4 (formerly ADH7) contain a homodimer of subunit π, χ, and δ (μ), respectively. The amino acid sequences of ADH 1, 2, 3, and 4 are about 60% homologous [77]. The *K_m_* for ethanol increases in the order ADH 1, 2, 4, 3, with the values being around 1 mM, several tens of millimoles, several hundred millimoles, and several moles, respectively [13,16]. The insensitivity to pyrazoles, which are specific inhibitors of ADH, also increases in the same order [13,16]. Thus, among mammalian ADH isozymes, ADH3 has the highest *K_m_* for ethanol and the lowest sensitivity to pyrazoles, while ADH1 has the lowest *K_m_* and the highest sensitivity. Unlike other ADHs, which have limited tissue distributions, ADH3 is present in almost all mammalian tissues. Moreover, ADH3 exists in all biological kingdoms, while other mammalian ADHs appear first in vertebrates. So, ADH3 is considered to be the ancestor of all other ADHs [68].
